# CMV immune evasion and manipulation of the immune system with aging

**DOI:** 10.1007/s11357-017-9986-6

**Published:** 2017-06-24

**Authors:** Sarah E. Jackson, Anke Redeker, Ramon Arens, Debbie van Baarle, Sara P. H. van den Berg, Chris A. Benedict, Luka Čičin-Šain, Ann B. Hill, Mark R. Wills

**Affiliations:** 1 Department of Medicine, University of Cambridge, Box 157, Level 5, Addenbrooke’s Hospital, Hills Road, Cambridge, CB2 0QQ UK; 2 Department of Immunohematology and Blood Transfusion, Leiden University Medical Center, Leiden, The Netherlands; 3 Department Immune Mechanisms, Center for infectious Disease Control, National Institute of Public Health and the Environment, Bilthoven, The Netherlands; 4 Department Immunology, Laboratory of Translational Immunology, University Medical Center Utrecht, Utrecht, The Netherlands; 5 Division Immune Regulation, La Jolla Institute for Allergy and Immunology, La Jolla, CA 92037 USA; 6 Helmholtz Centre for Infection Research GmbH, Inhoffenstraße 7, 38124 Braunschweig, Germany; 7 Institute for Virology, Medical School Hannover, Carl-Neuberg Str. 1, 30625 Hannover, Germany; 8 German Centre for Infection Research, Partner Site Hannover/Braunschweig, Braunschweig, Germany; 9 Molecular Microbiology & Immunology Department, OHSU, Portland, OR USA

**Keywords:** Cytomegalovirus, Immune evasion, Aging, Immune manipulation

## Abstract

Human cytomegalovirus (HCMV) encodes numerous proteins and microRNAs that function to evade the immune response and allow the virus to replicate and disseminate in the face of a competent innate and acquired immune system. The establishment of a latent infection by CMV, which if completely quiescent at the level of viral gene expression would represent an ultimate in immune evasion strategies, is not sufficient for lifelong persistence and dissemination of the virus. CMV needs to reactivate and replicate in a lytic cycle of infection in order to disseminate further, which occurs in the face of a fully primed secondary immune response. Without reactivation, latency itself would be redundant for the virus. It is also becoming clear that latency is not a totally quiescent state, but is characterized by limited viral gene expression. Therefore, the virus also needs immune evasion strategies during latency. An effective immune response to CMV is required or viral replication will cause morbidity and ultimately mortality in the host. There is clearly a complex balance between virus immune evasion and host immune recognition over a lifetime. This poses the important question of whether long-term evasion or manipulation of the immune response driven by CMV is detrimental to health. In this meeting report, three groups used the murine model of CMV (MCMV) to examine if the contribution of the virus to immune senescence is set by the (i) initial viral inoculum, (ii) inflation of T cell responses, (iii) or the balance between functionally distinct effector CD4+ T cells. The work of other groups studying the CMV response in humans is discussed. Their work asks whether the ability to make immune responses to new antigens is compromised by (i) age and HCMV carriage, (ii) long-term exposure to HCMV giving rise to an overall immunosuppressive environment and increased levels of latent virus, or (iii) adapted virus mutants (used as potential vaccines) that have the capacity to elicit conventional and unconventional T cell responses.

## Introduction

### CMV immune evasion during lytic infection

It is clear that primary human cytomegalovirus (HCMV) infection elicits a series of robust cell-mediated immune responses initially by innate NK cells, followed by adaptive CD4+ and CD8+ T cells and B cell high avidity neutralizing antibodies (reviewed in Jackson et al. 2011). These responses are essential in controlling viral replication and dissemination as shown by primary infection in either the immune-naive or immunosuppressed. Here, uncontrolled virus replication leads to end organ disease and morbidity and if left uncontrolled, mortality (Carbone 2016; Chan and Logan 2017; Kagan and Hamprecht 2017). Primary HCMV infection has a profound effect on the human immune system, leaving a permanent signature in the form of phenotypically distinct T and NK cell subsets at high frequencies (discussed in the accompanying article by Souquette et al.). However, despite this robust host immune response, HCMV is never cleared after primary infection, but persists for the lifetime of the host. Crucial to this lifelong persistence is the ability of the virus to establish a latent infection, in which infected cells carry viral genome but with limited viral gene expression and the absence of production of new infectious virions (Sinclair 2008). Importantly, the virus in these latently infected cells has the capacity to sporadically reactivate, leading to further rounds of antigenic stimulation and secondary immune responses with the associated release of inflammatory mediators. These rounds of virus reactivation and immune system stimulation can potentially drive further immune cell differentiation and increase the frequency of CMV-specific T cells. The latter phenomenon has been termed “memory inflation” in the murine CMV (MCMV) model and is characteristic of CMV infection (O'Hara et al. 2012).

Paradoxically, HCMV is recognized as a paradigm for a human pathogen encoding numerous viral immune evasion proteins and microRNAs (miRNAs), which are able to orchestrate a sophisticated array of immune evasion mechanisms. The mechanisms that modulate the infected cellular environment to limit immune recognition are most extensively expressed during lytic infection, but it is starting to become clear that viral gene activity during latency also acts to prevent immune clearance.

During lytic infection, specific genes encoded by HCMV can directly modulate innate/intrinsic immune responses such as the interferon responses (Amsler et al. 2013) as well as both intrinsic and extrinsic apoptosis pathways (Fliss and Brune 2012). The virus encodes proteins that act as receptors for host inflammatory cytokines, thereby reducing localized cytokine effectiveness by acting as cytokine sinks (McSharry et al. 2012). HCMV encodes a number of viral homologs of cytokines like UL146 (IL-8 like) and UL111a (vIL-10), an immunosuppressive IL-10 homolog (Cheung et al. 2009; Stern and Slobedman 2008). IL-10 is a powerful inhibitor of Th1 cytokines (such as IFN-γ and IL-2) and also inhibits inflammatory cytokine production from monocytes and macrophages which results in a decrease in surface MHC class II expression and a reduction of antigen presentation to CD4+ T cells (Opal and DePalo 2000).

HCMV interference with normal MHC class I expression to modulate CD8+ T cell recognition (see below) would lead to reduced inhibitory signaling and NK cell recognition of infected cells if additional viral mechanisms were not utilized. It is of little surprise then that a substantial number of HCMV proteins target multiple different pathways in order to modulate NK cell recognition. These include preventing HCMV-infected cells from expressing ligands at their cell surface that engage activating NK cell receptors, such as MIC A/B and the ULBP family which bind activating NKG2D; notably, both MCMV (Lisnic et al. 2015) and rhesus CMV (RhCMV) (Sturgill et al. 2016) also target NKG2D ligands. The virus also promotes the expression of ligands that are able to engage inhibitory receptors. For example, CMV encodes a viral MHC class I-like molecule, UL18, which binds the LIR-1 inhibitory NK cell receptor (only expressed by a subset of NK cells) and via a peptide processed from the viral UL40 protein is able to promote cell surface expression of HLA-E which binds the inhibitory heterodimer CD94/NKG2A. HCMV also targets PVR and Nectin-2, important ligands of activating receptors DNAM-1 and Tactile using UL141. UL141 of HCMV is further able to directly bind and inhibit expression of the TRAIL death receptors (Nemcovicova et al. 2013; Smith et al. 2013), a second avenue to dampen NK-mediated killing and a mechanism also employed by MCMV via m166 (Verma et al. 2014), also reviewed in Lisnic et al. 2015 and Wilkinson et al. 2008.

A number of HCMV-encoded genes expressed during lytic infection can interfere with both MHC class I and II restricted antigen processing and presentation. Proteins encoded within the US2-11 gene cluster target MHC class I and II molecules for retention within the endoplasmic reticulum (ER), re-direct MHC for degradation, and inhibit normal loading of peptides onto MHC class I. It is again noteworthy that MCMV and RhCMV have either viral homologs or other viral proteins which target normal MHC class I processing and antigen presentation (Lemmermann et al. 2012; Pande et al. 2005). In addition, two structural tegument proteins introduced into cells immediately upon infection, UL82 (pp71) and UL83 (pp65), interfere with ER egress of MHC I to the Golgi apparatus and with viral peptide generation, respectively (reviewed by Noriega et al. 2012). As discussed earlier, while HCMV downregulates classic HLA-A and HLA-B MHC I alleles, it has a mechanism that preserves HLA-E expression, and it is noteworthy that in RhCMV vaccine vectors, peptide presentation via non-classical HLA-E molecules is the basis for protective T cell responses, and work discussed by Ann Hill later investigates to what extent this is reflected in HCMV.

### Immune evasion during latency

Virus-encoded miRNAs which are expressed during the lytic viral cycle potentially provide an ideal mechanism to mediate immune evasion in latently infected cells. Recent evidence shows that many, if not all, of the HCMV-encoded miRNAs are expressed during latency (Lau et al. 2016); miRNAs have a major advantage in that they are not antigenic as far as T cell recognition is concerned. At least five miRNAs have been shown to target components of the immune system during lytic infection. miR-UL112.1 targets MICB, a ligand for NK cell activating receptor NKG2D (Stern-Ginossar et al. 2007); miR-US4.1 downregulates ERAP-1, an amino-peptidase which trims peptides for presentation by MHC class I, leading to decreased HCMV-specific CD8+ CTL recognition of HCMV-infected cells (Kim et al. 2011); miR-UL148D targets the chemokine CCL5 (RANTES), which is a T cell chemoattractant (Kim et al. 2012); miR-US25-2-3p targets inhibitors of TIMP3, leading to increased MICA shedding (also decreasing NK cell activation via NKG2D); and miRUl112-3p targets the Toll receptor TLR2 (reviewed in Piedade and Azevedo-Pereira 2016).

Three HCMV proteins, US28, UL111A, and UL144, that are expressed in latently infected cells (Goodrum 2016) have known immune evasion functions during lytic infection. US28 is a G protein-coupled receptor that can bind many chemokines including CCL5, MCP-1, MCP-3, MIP1-α, MIP1-β, and fractalkine. Binding of these chemokines to US28 results in their internalization and it has been suggested that US28 functions as a “chemokine sink,” reducing the local concentration of these inflammatory and chemotactic cytokines during lytic infection (Bodaghi et al. 1998). A splice product of UL111A is expressed during latency and encodes a viral IL-10 homolog (LAvIL-10) which acts to downregulate MHC class II expression on experimental latently infected myeloid cells modulating CD4+ T cell recognition (Cheung et al. 2009; Jenkins et al. 2004; Jenkins et al. 2008). UL144 has sequence similarity with members of the tumor necrosis factor receptor superfamily (TNFRSF) (Benedict et al. 1999; Locksley et al. 2001; Ware 2003) as well as the herpes simplex virus entry mediator (HVEM). UL144 has two putative immune evasion functions; the ecto-domain has been shown to interact with B and T lymphocyte attenuator (BTLA)-inhibiting T cell proliferation in vitro (Cheung et al. 2005) and the intracellular domain signals via NF-κB, TRAF6, and TRIM23 to induce production of the chemokine CCL22 which acts as a Th2 type chemoattractant, possibly subverting the Th1 immune response (Poole et al. 2008; Poole et al. 2009; Poole et al. 2006).

Analysis of the secretome of cellular proteins produced by experimental latently infected CD34+ progenitor cells has identified a number of proteins, including CCL8, cIL-10, and TGF-β, which are involved in immune response regulation and chemotaxis (Mason et al. 2012). CCL8 recruits CD4+ T cells to latently infected cells, where the CD4+ T cell encounters the substantial levels of the immune suppressive cytokines cIL-10 and TGF-β produced by the infected cell. IL-10 and TGF-β inhibit anti-viral IFN-γ and TNF-α cytokine secretion and cytotoxic effector functions of HCMV-specific Th1 CD4+ T cells. In addition, uninfected bystander CD34+ cells were induced to secrete TGF-β and cIL-10 by the latent CMV-infected cell secretome. This evidence strongly suggests that the microenvironment around latently infected CD34+ cells is immunosuppressive to T cell function.

Viral proteins expressed during latency have important roles in maintaining latency and preventing T cell recognition. As such are they themselves targets for T cells? LUNA, UL138, US28, and LAvIL-10 have been shown to be recognized by T cells; however, the predominant response was mediated by CD4+ T cells, many of which were not classic IFN-γ Th1 cells but secrete the immunomodulatory cytokines cIL-10 and TGF-β (Mason et al. 2013). In MCMV infection, IL-10 producing CD4+ T cells have been isolated from salivary glands, and in IL-10 KO mice or following IL-10R blockage, the latent MCMV load is reduced. This is consistent with the view that cytomegalovirus uses regulatory T cells to prevent latently infected cells from being recognized by the immune system (Humphreys et al. 2007; Jones et al. 2010). Chris Benedict describes later in this review the current evidence for the immunomodulatory role of the IL-10 axis and its impact on CD4+ T cell responses in MCMV infections.

### Evidence for CMV manipulation of the immune system with aging

As humans age, there are alterations to the immune response that can be potentially detrimental to health. For example, there is evidence from many studies that people aged over 65 years are more susceptible to infectious diseases (reviewed in Denkinger et al. 2015; Kline and Bowdish 2016). However, in the healthy elderly, overt disease caused by CMV is rarely seen (Stowe et al. 2007), despite the dual manipulation of the immune response by the virus and the effects of natural aging. There is some limited evidence in humans that modulation of the immune response by HCMV in the aged is occurring, potentially resulting in less effective control of viral replication following reactivation of virus. This hypothesis was suggested by the following observations: (i) CMV DNA was detectable in the urine of old but not young donors (Stowe et al. 2007), (ii) an increase in viral DNA has been detected in the blood of older donors in a Japanese cohort (Furui et al. 2013), and (iii) a UK study detected a significant increase in latent CMV genomes (in peripheral blood CD14+ monocytes) in people aged over 70 years (Parry et al. 2016). To counteract this evidence, there have been studies of aged CMV-positive people, which have been unable to detect viral DNA in the blood (Stowe et al. 2007; Vescovini et al. 2004). Wills and Jackson present in this review some experimental evidence, which suggests there may indeed be a slight loss of control of viral replication in the aged compared to younger individuals.

There is however evidence that CMV infection might be altering the balance of immune responses in aging, from the strong association of CMV seropositivity with increased mortality from cardiovascular disease (Gkrania-Klotsas et al. 2012; Olson et al. 2013; Savva et al. 2013; Simanek et al. 2011; Spyridopoulos et al. 2016). It has been proposed that the association of CMV infection with cardiovascular disease may be a direct result of pathology caused by the large expansion of CD4+ CD28-negative T cell populations (Broadley et al. 2017) commonly seen in CMV-infected individuals (Weltevrede et al. 2016).

As previously discussed, HCMV infection modulates the hosts IL-10 pathway, both directly through expression of viral-encoded IL-10 homologs (Christiaansen et al. 2015) and indirectly by altering the secretome of the infected cell, in addition to generating cellular IL-10-secreting CMV-specific CD4+ T cells. The production of cellular IL-10 in response to HCMV antigenic stimulation by CD4+ T cells has been observed in multiple studies (Clement et al. 2016; Jackson et al. 2017a; Mason et al. 2013; Schwele et al. 2012). Production of IL-10 is a characteristic feature of regulatory T cells (Tregs) (Zhu et al. 2010). CMV-specific Tregs in humans have been identified in a number of studies using various methods of Treg phenotyping (Clement et al. 2016; Derhovanessian et al. 2015; Mason et al. 2013; Schwele et al. 2012; Terrazzini et al. 2014; Tovar-Salazar et al. 2010). The Schwele et al. study demonstrated that the generation of CMV-specific inducible Tregs (iTregs) is most likely to be related to frequent episodes of antigenic reactivation (Schwele et al. 2012), a further method that the virus can use to modulate T cell populations. Suppressive CMV-specific iTregs have also been identified in the expanded CD4+ CD28-CD27 population (Tovar-Salazar et al. 2010) and their frequency increased with age and was associated with vascular pathology along with expanded CMV-specific CD8+ effector T cell populations (Terrazzini et al. 2014). In the murine model, it has been demonstrated that IL-10 secretion and Tregs reduce clearance of the virus and increase persistence (Clement et al. 2016; Jost et al. 2014). This phenomenon has been demonstrated with CD4+ T cells resident in the salivary glands (Humphreys et al. 2007) and the liver (Popovic et al. 2017).

While clinical studies in humans have allowed for important insights into pathologies associated with HCMV persistence, by their very nature, they cannot define if CMV is the cause of (i) pathology, (ii) a consequence of underlying predispositions for associated pathologies, or (iii) merely an accidental and adventitious correlation. Defining cause-effect relationships in HCMV immunology requires an experimental method and such studies are hindered by the strict species specificity of this virus. Therefore, studies in animal experimental models have relied on CMV ortholog viruses in their natural host species. While data from animal models are not necessarily a direct representation of natural events, this caveat holds for any dataset generated in a model, whether animal, experimental, or mathematical.

Using the mouse model, it has been shown that CMV infection results in memory inflation of T cell populations specific to particular MCMV protein epitopes (Karrer et al. 2003) and is another example of the impact CMV infection can have on host immune responses over a lifetime. Despite the dominance of a restricted clonal MCMV-specific T cell populations as a proportion of total T cell compartment (Karrer et al. 2003), it has been clearly shown that the expanded memory T cells are still functional and dynamic (Snyder et al. 2008). These cells can be replenished to similar expanded levels following depletion (Sims et al. 2015) and the inflation of this population can occur, although less pronounced, in the absence of reactivating or replicating virus (Snyder et al. 2011). The lessons that can be learnt from the manipulation of the immune response by MCMV infection in the mouse and its impact on aged T cell responses are discussed later in this review by Ramon Arens and Anke Redeker, and Luka Cicin-Sain.

Previously, it has been suggested that the expansion of CMV-specific CD8+ and CD4+ T cells that have been reported in some human aging studies is also evidence of memory inflation within HCMV infection (studies reviewed in Weltevrede et al. 2016). However, unlike experiments in the murine model, most of the evidence for memory inflation in humans comes from cross-sectional age studies (Komatsu et al. 2003), rather than longitudinal studies tracking the size of CMV-specific CD8+ and CD4+ T cells within particular individuals over many years. It has been reported in individuals that as much as 10% of the total CD8+ T cell compartment (Sylwester et al. 2005) and 5% of the total CD4+ T cell compartment (Pourgheysari et al. 2007) are composed of CMV-specific responses. The expansion in size of the CMV-specific T cell response in humans is at the expense of T cell responses to other antigens. This hypothesis has been used previously to explain the observations from a number of studies where there is an increase in all-cause mortality and susceptibility to new infections in elderly CMV seropositive donors (Hadrup et al. 2006; Olsson et al. 2001; Ouyang et al. 2004; Strindhall et al. 2013; Wikby et al. 2002). However, there have been a number of studies that dispute this hypothesis. It has been shown that poor responses to vaccines in older people are irrespective of CMV seropositivity (Furman et al. 2015) and the ability of older people to respond to novel infections is not impaired by an existing CMV infection (Lelic et al. 2012; Schulz et al. 2015). Debbie van Baarle and Sara van den Berg discuss the impact of CMV infection and consequential immune modulation on the ability to respond to neo-antigens in old age further later in this review.

## Current research perspectives

### The impact of aging on IL-10-secreting HCMV latent antigen-specific T cells and latent viral load (Sarah Jackson and Mark Wills)

Human cytomegalovirus (HCMV) infection and the periodic re-activation of latent virus are generally well controlled by T cell responses in healthy people (Crough and Khanna 2009; Jackson et al. 2011). Within older donors, although overt disease caused by HCMV is rarely seen (Stowe et al. 2007), there is some evidence discussed in the introduction that suggests there may be immunomodulation of the immune response in the elderly, potentially resulting in low-level viral replication and dissemination without causing overt disease. Latent CMV infection is characterized by limited viral transcription, with only a handful of viral genes (e.g., UL138, LUNA, US28, UL111A, and UL144) being transcribed (Goodrum 2016). Previously, we have reported that there are IFN-γ CD4+ T cell responses to UL138 and LUNA proteins that are cytotoxic and there are also cellular IL-10 responses by CD4+ T cells in response to these latent associated proteins (Mason et al. 2013). Therefore, IL-10 is a candidate to mediate immunomodulation of the CMV-specific immune response during aging (Wills et al. 2015). To address the question of whether long-term carriage of HCMV changes the proportions of IL-10 and IFN-γ secreting HCMV-specific T cell populations, Jackson and Wills recruited a large cohort of CMV seropositive donors aged 20–80 years in association with the Cambridge Bioresource. We correlated the CD8+ and CD4+ T cell responses with 11 HCMV proteins (5 latent ORFs and pp65, pp71, gB, IE1, IE2, and US3) with age, HCMV IgG levels, latent HCMV load in CD14+ monocytes, lytic HCMV DNA copies in whole blood, and absolute T cell population counts from whole blood.

The recruited donor cohort had a significant decline in total and naïve CD8+ and CD4+ T cell numbers, which has been reported previously in different aging studies (summarized in Weltevrede et al. 2016). The impact of CMV infection resulted in increased numbers of differentiated CD8+ and CD4+ T cell populations, which has often been described (Weltevrede et al. 2016); however, this phenomenon was irrespective of donor age in this cohort. Both CD4+ and CD8+ T cells responded to stimulation with all 11 HCMV proteins, and there was no accumulation of specific IFN-γ T cell responses with increasing age; the CD4+ T cell IL-10 response was less frequently observed; however, overall, the breadth and magnitude of the IL-10 response to HCMV proteins remains stable regardless of donor age. IL-10-secreted CD4+ T cell responses were predominantly to latency-associated proteins (LUNA, UL138, US28, and vIL-10), although the lytic-expressed proteins pp71 and US3 also triggered a number of donors to produce a CD4+ T cell-specific IL-10 response. Within this cohort, CMV IgG levels remained stable and there was no inversion in the CD4:CD8 ratio with increasing donor age. Measurement of HCMV viral copy numbers in CD14+ monocytes, a known cellular site of latent CMV carriage (Reeves et al. 2005), did not reveal any increase in viral levels in older donors, in contrast to a previous UK study (Parry et al. 2016). Importantly, however, we did see a significant correlation between increased latent viral copies and the breadth and magnitude of the IFN-γ CD8+ T cell response. Our hypothesis is that a larger latent HCMV reservoir may result in more frequent HCMV re-activation and dissemination events, which consequently lead to expansion of CMV-specific T cell responses (and potentially re-seeding of the latent pool). We did not detect HCMV DNA in the blood of 104/105 HCMV seropositive donors included in the study; interestingly, the one donor who did have low levels of detectable HCMV DNA (274 copies/ml whole blood) was an aged male donor who had an inverted CD4:CD8 ratio and an above average number of highly differentiated CD8+ T cells. This individual result is supportive of the hypothesis discussed in the introductory section, i.e., the elderly may not be as able to control CMV replication as adequately as the young, possibly due to manipulation of the immune response by the virus. This donor cohort was recruited on the basis of HCMV serostatus and those suffering from immune-altering illnesses or treatment for these conditions (e.g., cancer, autoimmune disorders, and systemic steroid treatment) were excluded from the study. This stringent recruitment criterion may have resulted in less healthy older donors, who may have lost control of CMV replication to some extent resulting in low-level viral dissemination, not being included in our study and potentially skewing our observations (Jackson et al. 2017b).

It is also important to assess the functional capacity of the CMV-specific T cells in response to the virus directly; again, this approach may reveal defects in the immune response to CMV in the elderly which are not apparent in other population studies. In order to address this problem, we have developed a viral dissemination assay which we have used to interrogate the capacity of HCMV-specific CD8+ (Jackson et al. 2014) and CD4+ T cells (Jackson et al. 2017a) to control the spread of HCMV in vitro. We have demonstrated that purified CD8+ and CD4+ T cell populations when isolated directly ex vivo are able to prevent the spread of the a GFP-tagged clinical CMV isolate. To date, we have only been able to use this methodology with a limited number of donors aged over 65 years; however, in the CD8+ T cell study, the evidence we do have suggests that the older donors were capable of controlling viral spread, but less efficiently compared to younger donors (Jackson et al. 2014). This observation is also supported by our CD4+ T cell work, where the older donor included in the study demonstrated a distinct loss of control of viral dissemination at low T cell to infected cell ratios, which was not seen in the younger donors (Jackson et al. 2017b). These preliminary results suggest that the viral dissemination assay will enable a more comprehensive insight in to understanding if there is a diminution in the effectiveness of CMV-specific T cells in the elderly in the context of an active viral infection, where the full armory of the virus immune evasion and manipulation mechanisms are expressed.

### Fibroblast-adapted HCMV vaccines elicit conventional CD8+ T cell responses in contrast to RhCMV vaccines (Ann Hill)

Studies using CMV as a vaccine vector in the monkey model of AIDS have uncovered a highly unusual aspect of CMV immunology. After serial passage in vitro, HCMV isolates become adapted to the fibroblasts they are grown in by losing the ability to form a functional pentameric complex of glycoproteins that is needed for entry into most non-fibroblast cells. When Louis Picker and colleagues developed their rhesus (Rh) CMV vaccine vector, they used Peter Barry’s RhCMV BAC, which was derived from a fibroblast-adapted RhCMV (strain 68.1) and was pentameric complex-defective (Hansen et al. 2009; Yue et al. 2016). RhCMV expressing proteins from simian immunodeficiency virus (SIV) has provided impressive protection from a virulent SIV challenge, and the strategy is being very actively pursued to develop a vaccine for human AIDS (Hansen et al. 2013a). Studies to elucidate the immunological mechanism of the RhCMV-vectored vaccine’s efficacy have yielded startling results: most of the antigen-specific CD8+ T cells elicited by the vaccine are completely unconventional, being restricted either by MHC II or the rhesus equivalent of HLA-E (Hansen et al. 2013b). Further studies showed that the unconventional responses were only elicited by RhCMV lacking a functional pentameric complex: restoring the pentameric complex led to the generation of conventional, i.e., classical MHC1-restricted, responses. These unconventional responses are believed to be responsible for the vaccine’s efficacy against SIV. If that is the case, it is important to know whether the strategy could be translated into a human vaccine.

CMV’s strict species specificity means that translating the monkey results to humans requires a shift in not only SIV to HIV antigens but also of the vector, from RhCMV to HCMV. Additionally, rhesus and human immune systems have some differences. Most remarkable is the rhesus MHC complex, which is extremely polymorphic and polygenic. Up to 22 separate classical MHC I isoforms are simultaneously expressed, contrasting with up to 6 in humans or mice (de Groot et al. 2015). The MHCII loci are similarly polygenic and polymorphic in monkeys. It could be that the unconventional responses to RhCMV result from a distorted CD8+ T cell repertoire that has arisen because of excessive negative selection by this highly diverse MHC. However, it should be noted that all other T cell responses studied in macaques have been conventional, including those elicited by wild-type, i.e., pentameric complex sufficient, RhCMV. Hence, it is likely that immune system manipulation by CMV itself is responsible for these responses, and that they would also be seen in humans.

As a first attempt to look at this question, we studied the CD8+ T cell responses to fibroblast-adapted HCMV vaccines (Murray et al. 2017). Stuart Adler’s group had conducted a dose escalation phase I clinical study of four vaccines that are chimeras between Towne and Toledo strains (Adler et al. 2016). All share the same defect in the pentameric complex genes and have impaired cellular tropism in vitro for epithelial, endothelial, and macrophage cells. We were able to study the CD8+ T cell responses in six subjects. All subjects responded to HCMV IE1, and the typical pattern of immunodominant responses to one to two epitopes was seen. This contrasts with the responses reported in monkeys, which responded to an average of 36 epitopes in RhCMV IE1. Most responses in the human subjects were mapped to a minimal epitope, and HLA restriction was mapped for eight epitopes. Each epitope that could be mapped was conventionally MHC I restricted, and two subjects responded to previously identified HCMV epitopes. Antibody blockade and use of HLA-E transfectants provided no evidence to suggest that the remaining responses were unconventional.

The response to these pentameric complex-deficient HCMV vaccines thus appears to be predominantly or completely conventional in human subjects. This could be a result of differences in human and monkey immune systems. However, it should be noted that although both are pentameric complex deficient, the Towne-Toledo chimeras and RhCMV 68-1 differ in the genetic mutations that result in the functional deficiency. It will be important to understand the viral mechanism for eliciting the unconventional responses to have the best chance of eliciting the same responses in humans, should they prove to be important for the vaccine’s efficacy.

### The effect of CMV on the response to influenza virus vaccination (Debbie van Baarle and Sara van den Berg)

Immunosenescence is the age-related deterioration of immunocompetence which is reflected in a poorer response to (new) antigens and leads to increased susceptibility of elderly to infection and lower response to vaccination. HCMV infection has been shown to enhance deleterious age-associated changes in immunity and may thereby contribute to poor responses to vaccinations. Indeed, it has been shown that HCMV is part of the immune risk profile (IRP), which is associated with all cause of death (Olsson et al. 2001). Based on a systematic review of studies performed between 2004 and 2014 on the role of HCMV persistence on T cell immunosenescence in people aged 50 and older, we observed that CMV mainly seems to enhance immunosenescence through increasing the levels of the highly differentiated effector memory (TEM) and CD45RA-expressing effector memory (TEMRA) cells in the CD8+ and CD4+ T cell pools (Weltevrede et al. 2016), although an elegant study (Wertheimer et al. 2014) suggested also a decline in CD4+ T cells in HCMV-positive individuals. Furthermore, CMV infection was also shown to be associated with intrinsic B cell defects (Frasca et al. 2015). How these changes may affect responses to other infections or vaccinations and whether these shifts within the T cell compartments in HCMV-seropositive elderly are related to susceptibility to infectious diseases remain to be fully investigated.

**Fig. 1:**
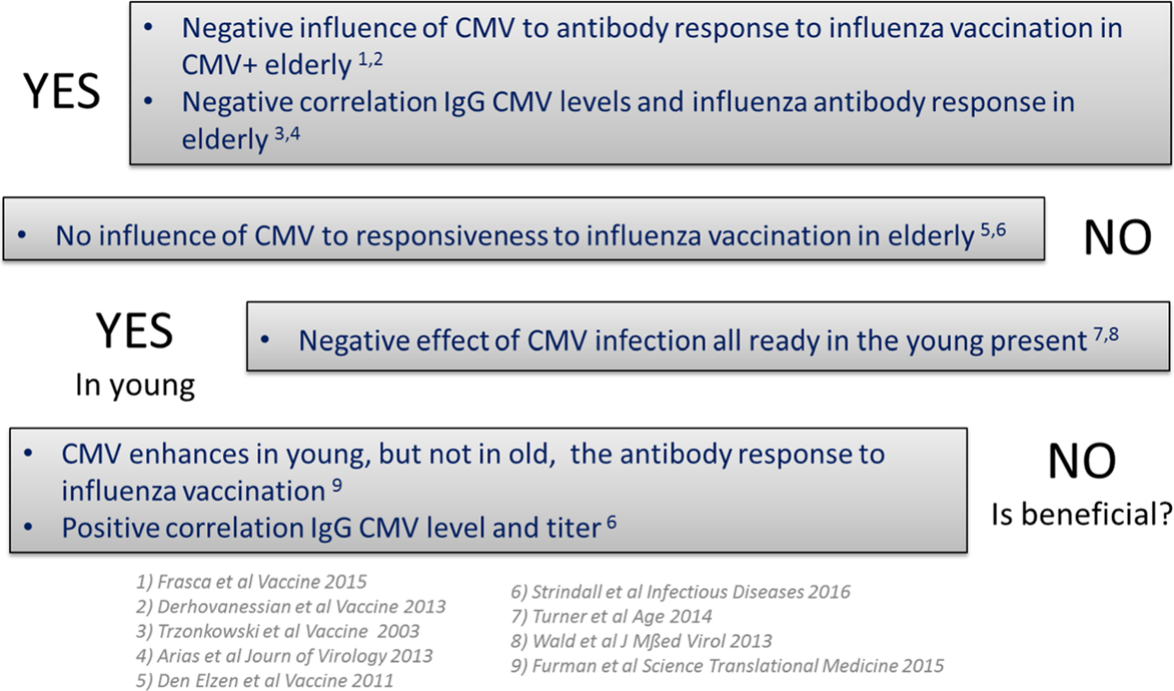
Overview of studies performed and their findings on the role of CMV infection on the immune/antibody response to influenza vaccination. Overview of Alonso Arias et al. 2013, den Elzen et al. 2011, Derhovanessian et al. 2013, Frasca et al. 2015, Furman et al. 2015, Strindhall et al. 2016, Trzonkowski et al. 2003, Turner et al. 2014, and Wald et al. 2013

In humans, in whom the effect of HCMV has especially been studied in the context of influenza virus vaccination which is shown to lead to less protection in older individuals, the data is less clear (see box/Fig. 1). Four studies suggest a negative effect of HCMV to antibody responses to flu vaccination on elderly (Alonso Arias et al. 2013; Derhovanessian et al. 2014; Frasca et al. 2015; Trzonkowski et al. 2003). This negative effect of HCMV was also found to be present in young individuals in two additional publications (Turner et al. 2014; Wald et al. 2013). However, two studies observed no effect of HCMV on influenza antibody responses in elderly (den Elzen et al. 2011; Haq et al. 2016), one of which observed a positive correlation between IgG HCMV levels and the influenza antibody titer, suggesting a beneficial effect of HCMV. Moreover, a recent paper by Furman et al. observed an enhancing effect of HCMV on the antibody response to influenza in young, but not in old individuals (Furman et al. 2015). These discrepancies in the literature may result from differences in pre-existing immunity to the seasonal influenza vaccination in participants. We hypothesize, therefore, as shown in HCMV models in mice (Cicin-Sain et al. 2012; Mekker et al. 2012) that CMV infection will have a larger impact on/impair the antibody response to neo-antigen influenza vaccinations.

In our study, focusing on middle-aged individuals (*n* = 287), of whom 60% were HCMV-seropositive and who were vaccinated with the novel pandemic influenza A strain (pH1N1) vaccine during the 2009 influenza pandemic, we found no effect of HCMV infection on antibody response to influenza vaccination, despite the fact that we do observe an age-related effect on influenza antibody responses in this group (S. P. H. van den Berg, manuscript in preparation). As detectable titers to the novel pandemic influenza strain were present before vaccination in 23% of the individuals, this still may have contributed to the lack of effect of CMV on influenza antibody responses. However, correction for pre-titers and other potential confounding factors like age, sex, and previous vaccination did not change the observed result. Furthermore, our data may even suggest a beneficial/positive effect of CMV infection on the protection rate after influenza vaccination in these adults. These data add to the discussion on the role of CMV infection on vaccination responses and suggest a scenario in which CMV infection may be beneficial during the first decades of life, but this effect may be lost upon aging.

In mouse models of MCMV infection in which MCMV-infected mice are challenged with neo-antigens, data are clear and specific defects in immunity to newly incoming pathogens have been reported leading to lower protection against these new infections (see Ramon Arens and Anke Redeker below). In these studies MCMV was shown to have effect mainly on the T cell compartment but effects on the antibody response were also observed. Prior MCMV infection was shown to result in significantly reduced (Cicin-Sain et al. 2012) or remodeled (Smithey et al. 2012) CD8+ T cell responses to challenging infections and elevated viral loads (Mekker et al. 2012). As HCMV mainly impacts the T cell repertoire and the T cell response plays a bigger role in cross-reactive immunity towards different influenza strains as well as vaccine-induced protection in later life, the question is whether we can find evidence for decreased cellular immune responses and whether previous HCMV infection would affect immune protection after vaccination in humans of different ages. A recent study shed first light on this by showing that CMV seropositivity was associated with a decline in Granzyme B responses to influenza and may predict increased susceptibility to influenza in older adults (Haq et al. 2016). In contrast, Theeten et al. show a positive relation between cellular IFN-γ and Granzyme B responses to CMV-pp65 antigen and influenza N1 antigen (Theeten et al. 2016). Thus, with regard to the effect of CMV on cellular immune responses to influenza in humans, the jury is still out.

### Impact of the infectious dose of CMV on the severity of immune senescence (Ramon Arens and Anke Redeker)

The controversies with respect to the role of CMV in immunosenescence including the impact of CMV infection on vaccines and encountering other pathogens as discussed by van Baarle and van den Berg above might relate to the differences in the immunological response rate to HCMV, which varies significantly. In the human population, the percentages of CMV-specific T cells occupying the memory T cell compartment are highly variable (ranges from 0.01 to 50%) (Vescovini et al. 2014). The average HCMV-specific memory T cell response is ~10% of the total memory T cell compartment and is compared to other infections, extraordinarily high (Sylwester et al. 2005). In old age, it has been estimated that the HCMV-specific memory T cell pool can occupy up to 50% of the total memory compartment (Vescovini et al. 2007). The high variation in the T cell response to CMV is likely related to the significant difference in the quantity of CMV found in bodily fluids such as breast milk, saliva, and urine (10^1^–10^5^ copies/μl), causing horizontal transmission of CMV (Cope et al. 1997; Kouri et al. 2010).

The mouse studies that have been performed previously, wherein the impact of long-term CMV infection on host immunity was investigated (Cicin-Sain et al. 2012; Mekker et al. 2012; Smithey et al. 2012), showed a relationship between CMV positivity and immune senescence. Specifically, these studies demonstrated that long-term latent CMV infection induces high numbers of effector-type like memory CD8^+^ T cells reactive to CMV, while newly generated CD8^+^ T cell responses to heterologous viral infections were diminished. However, this was not followed by an increase in mortality from heterologous viral infection (Marandu et al. 2015). This is in contrast with the controversial data observed in humans. However, these mouse studies used relatively high doses of virus for infection leading to high CMV-specific T cell responses. Hence, the diversity in the HCMV-specific immune response as seen in humans was not taken into account. Previously, we have shown that the degree of accumulation and phenotype of inflationary CMV-specific CD8^+^ T cells corresponds to the size of the initial infectious dose (Redeker et al. 2014). To gain insight into the controversial results with respect to the possible contribution of CMV to immune senescence in humans, and whether this is related to the large variance in the frequency, phenotype, and accumulation of CMV-specific memory T cells, we performed a highly controlled prospective study. We infected mice with different inoculum dosages of MCMV and investigated longitudinally the influence of lifelong CMV infection on alterations within the peripheral T cell pool. Our results show that the initial viral inoculum determines the degree of CMV-induced immune alterations in lifelong infection. A heightened MCMV infection resulted in reduced frequencies of naive circulating CD8^+^ T cells (CD44^low^CD62L^+^KLRG1^−^) and, on the other hand, increased the accumulation of effector memory (EM; CD44^high^CD62L^−^KLRG1^+^) CD8^+^ T cells. A remarkable correlation becomes apparent between the magnitude of the CMV-specific CD8+ T cell response and the EM phenotype of these cells. Basically, the higher the magnitude of response, the higher the frequency of the EM-like CD8+ T cells. Importantly, these correlations between the CMV-specific CD8+ T cell response and memory phenotype were also apparent in CMV-infected humans.

To specifically assess the role of the CMV inoculum size on the development of heterologous anti-viral immunity, aged MCMV-infected mice received a challenge with lymphocytic choriomeningitis virus (LCMV). Strikingly, mainly in the mice that were infected with the highest dose of MCMV, the LCMV-specific CD8^+^ T cell expansion was impaired. The latter correlated with a reduced activation status of the LCMV-specific CD8+ T cells, and importantly, the control of LCMV infection was hampered. This detrimental effect of high-dose infection was not observed in young mice, where even a positive effect was apparent. Thus, only in the high-dose-infected aged mouse the control of heterologous infection was found to be impaired. This suggests that predominantly in aged individuals with pronounced HCMV-induced perturbations, detrimental effects for these persons can be contributed to CMV while in persons with low HCMV-specific responses, negligible effects of CMV are present. Instead, in young persons, HCMV may even have beneficial effects. To settle these statements, additional prospective longitudinal studies are necessary that incorporate stratification based on the quantity and quality of the HCMV response in each individual.

### CD4 T cell control of CMV persistence during initial infection: how does it impact viral shaping of the immune system over a lifetime? (Chris Benedict)

As discussed above, HCMV-specific CD4+ T cells can secrete a variety of cytokines, producing some more frequently when they encounter their cognate antigens expressed by latently infected cells (e.g., IL-10). However, it is very challenging to assemble cohorts to assess T cell effector and memory responses during primary HCMV infection, given it is largely asymptomatic in healthy people. Therefore, most studies have measured the function and phenotype of these cells in persons already infected for many years/decades, long after the acute and high-level persistent replication phases of infection have been controlled. As the mouse provides a tractable model to study all three phases of CMV infection (acute, persistence, and latency), and T cells induced by MCMV show similar phenotypes and functions to that of HCMV, we set out several years ago to identify the first MCMV epitope-specific CD4+ T cells, characterizing 15 in C57BL/6 (B6) mice (I-Ab restricted) (Arens et al. 2008) and two in BALB/c (I-Ad restricted) (Verma et al. 2015). Our recent construction of several MHC II tetramers has allowed the enrichment and characterization of these cells from lymphoid and non-lymphoid tissues, a necessity given they are present at markedly lower numbers than their CD8+ T cell counterparts (Moon et al. 2007).

There is no identifiable IL-10 sequence ortholog encoded by MCMV, unlike the primate CMVs; however, various host immune cell types produce IL-10 during both the acute and persistent phases of infection. CD4+ T cells secrete the majority of this IL-10 during MCMV persistence in the salivary glands of B6 mice (Clement et al. 2016), where high-level viral replication continues for several months. Studies performed a decade ago showed that signaling by the IL-10 receptor sustains MCMV replication at this mucosal site (Humphreys et al. 2007) and restricts the expansion of CD8+ and CD4+ T effector cells (Jones et al. 2010), with a similar role also reported for RhCMV vIL-10 in monkeys (Chang and Barry 2010). In turn, IFN-γ^+^ CD4+ Th1-like cells play a key role in the eventual resolution of MCMV persistence (Lucin et al. 1992; Walton et al. 2011), while their production of TNF appears unnecessary for this (Fleck et al. 1998; Walton et al. 2011). Consequently, the relative numbers of CD4+ T effector and memory cells that produce IFN-γ and IL-10 during the various phases of CMV infection likely form an axis that regulates the magnitude, duration, and reactivation of CMV in both mice and men.

It has been shown that the ability to mounting a robust, IFN-γ^+^ secreting HCMV-specific CD4+ T cell response correlates with reduced duration of persistent CMV replication in the kidneys of young children (Tu et al. 2004). Initial MCMV infection induces a strong IFN-γ + CD4+ T cell response, and these cells are present in the salivary gland within 1 week. However, they are not sufficient to resolve viral replication at this time of “early persistence,” due at least in part to the high levels of IL-10 that are commensurately produced. Intriguingly, our past studies identifying MCMV epitope-specific CD4+ T cells found that some responses did not expand until much later times following infection, after systemic replication was controlled and persistence had been established. It is interesting to postulate that these “late-rising” CD4+ T cells might display distinct anti-viral effector functions during viral persistence compared to their conventional counterparts, as they would likely differentiate in a unique inflammatory environment. In turn, it is possible that the duration of CMV persistence prior to latency establishment may influence the viral impact on immune aging. For example, a longer persistent replication phase may result in a higher initial “set point” of CMV-specific cellular responses and latent virus load, with these differences being amplified over a subsequent lifetime. Perhaps, this contributes to the dose-dependent impact of initial MCMV infection on the T cell compartment in old mice described by Arens and Redeker. Finally, how these two distinct populations of conventional and late-rising CD4+ T cells induced by MCMV might equate to the HCMV-specific CD4+ T memory cells seen by Wills and colleagues that preferentially recognize viral antigens expressed during the lytic and latent phase of infection is unclear, if they do at all, but it is an intriguing question.

### Immune evasion of memory inflation in the murine model (Luka Čičin-Šain)

The mouse model (MCMV) reflects the balance between the latent virus and the host immune system, where a hierarchical and redundant cellular immune response keeps CMV from reactivating (Polic et al. 1998). The continuous low-level transcription of viral genes (Kurz et al. 1999) results in the persistence of T cell infiltrates in the lungs of latently infected mice (Podlech et al. 2000), where immunodominant effector or effector memory T cells against epitopes transcribed in latency prevail (Holtappels et al. 2000). The expansion and persistence of T cells against immunodominant CMV antigens has been aptly named memory inflation (Karrer et al. 2003) and it was shown that it reflects T cell responses to HCMV (Komatsu et al. 2003). Importantly, memory inflation is an expansion of effector T cells against defined MCMV-specific epitopes, but the size of the effector T cell pool remains unaltered (Cicin-Sain et al. 2012), implying that memory inflation is a focus of CD8+ T cell responses on few immunodominant epitopes at the expense of subdominant ones, rather than a general expansion of T cell responses to cytomegalovirus. The size of the inflationary response depends on the latent transcriptional activity of the viral genes encoding the epitope (Dekhtiarenko et al. 2013) and on its availability for processing by the constitutive proteasome (Dekhtiarenko et al. 2016; Hutchinson et al. 2011), implying that CD8+ T cell re-stimulation may occur by antigens presented directly on latently infected cells. In fact, the expansion of MCMV-specific CD8+ T cells in memory inflation requires antigen presentation on non-hematopoietic cells (Seckert et al. 2011; Torti et al. 2011), likely from within the latently infected cell. It has remained somewhat controversial if this phenomenon depends on the viral evasion of the immune system, or if it occurred independently of it.

Akin to the HCMV situation, MCMV devotes a substantial part of its genome to the evasion of the immune system. It has developed multiple strategies to downregulate MHC I molecules from the surface of infected cells and thus avoid detection by CD8+ T cells (Wagner et al. 2002). However, an MCMV mutant lacking all viral genes known to interfere with MHC I surface presentation induced similar hierarchies of CD8+ T cell immunodominance as the wild-type MCMV, both in primary and in latent infection (Munks et al. 2007). This observation might be anticipated if one considers that the known MHC I downregulators m04, m06, and m152 are lytic viral genes that are unlikely to be expressed during virus latency. Surprisingly, another study showed that the MCMV lacking these three genes induces a weaker CD8+ T cell response (Bohm et al. 2008), arguing for a paradoxically improved priming and inflation state in the presence of immune evasion. The latter study showed that immune evasive genes protect the virus from the antiviral activity of CD8+ T cells, allowing it to proliferate longer and to higher titers in draining lymph nodes during primary infection (Bohm et al. 2008). Therefore, it was proposed that immune evasion enhances viral replication, antigen availability for priming, and consequently, the size of the inflationary response. The conditions of primary infection and inoculum define the size of the latent reservoir (Reddehase et al. 1994) and of the inflationary response (Redeker et al. 2014). In particular, the latent MCMV burden in the spleen, but not in in the lungs, is associated with a more pronounced inflationary response to MCMV (Oduro et al. 2016). Consequently, by improving viral fitness in the primary infection and by increasing the number of cells carrying latent viral genomes, immune evasive genes may indirectly affect the size of the inflationary response, although they are not expressed at the time of latency and T cell expansion.

This does not exclude possible additional effects of viral genes at the time of latency. The CMV genome is the largest known among mammalian viruses and the function of numerous viral genes remains unknown (Rawlinson et al. 1996). Beyond the viral genes that downregulate MHC I from cell surface, CMV expresses numerous additional genes which may interfere with CD8+ T cell priming, for instance those interfering with costimulatory signal 2 or 3 (Doring et al. 2014; Loewendorf et al. 2004). Therefore, it is likely that numerous additional genes interfere with the immune response and it cannot be excluded that their activity may influence the size and the quality of the inflationary T cell response. A shotgun approach to the study of immune evasion by targeted deletion of large genomic regions rich in immune evasive genes yielded an MCMV variant lacking 34 viral genes in regions rich in immune evasive genes (Cicin-Sain et al. 2007). While this mutant induced inflationary responses that could not be distinguished from wild-type MCMV in size (Cicin-Sain et al. 2007), it cannot be excluded that other viral genes—including the essential ones—may affect the T cell responses. Therefore, future studies in models of in vivo infection may inform us if immune evasion acts actively during viral latency and if it defines the size and the functionality of the responding T cell pool.

## Future perspectives

The preceding summaries of current research work in both humans and mouse models go some way to assessing the impact that CMV infection and viral immune evasion may have on the immune response to CMV and the immune response more generally. However, there are still many unanswered questions, and the real impact of CMV manipulation of the immune response on the elderly remains to be fully ascertained. We propose that future studies may wish to consider the following issues:

- To what extent is the association of CMV infection with detrimental changes to the immune response in aging a co-incidence or a real phenomenon, and, if so, is the virus the direct causative agent or are the changes to the immune response a bystander effect of CMV infection? While this question is not straightforward to address in humans, due to the many unknowns in the CMV infection history of an individual subject, designing clearly defined population studies which measure multiple parameters (e.g., pertinent medical history, socio-economic background, country or region of birth, absolute immune cell counts, CMV-specific immune responses and quality/quantity of these—both T cell and immunoglobulins, CMV DNA quantification) will help going forward. Additionally, the adoption of similar strategies and measurements in geographically distinct donor cohorts will enable comprehensive intra-study comparison, thus increasing the overall power of the conclusions that can be derived.
- Is CMV infection an important comorbidity factor in human aging? To address this question will require comparison of the impact of CMV infection in less healthy elderly donor cohorts, e.g., does CMV infection in donors with cardiovascular-related health problems lead to less control viral reactivation and dissemination, compared to age-matched healthy people. This question could be asked in a number of different disease cohorts; however, in cases of cancer or auto-immune diseases (e.g., rheumatoid arthritis), the impact of these conditions on the immune response will have to be accounted for when interpreting the results.
- Does CMV-mediated modulation of the immune response in the elderly result in low-level viral reactivation and dissemination, that is below the level required to present as overt clinical disease but nevertheless may drive inflammatory pathologies? This will require that future studies incorporate a virological assessment of donors, with quantification of virions present (or not) in blood, saliva, and urine alongside measuring typical immunological parameters, could be highly informative.
- As illustrated in this review, the use of the mouse model has been very important in understanding the multiple consequences of CMV infection, immune evasion, and manipulation on the host immune response. However, this does not replace the need for studies to be performed in humans, particularly in the context of immune aging research. Extrapolating results from murine aging studies to humans requires consideration of a number of key differences: (i) the contribution from thymic output replenishing the peripheral T cell compartment is higher in mice than men (Appay and Sauce 2014); (ii) mouse telomeres are proportionally longer than humans; therefore, it is less likely that immune cells from aged mice will exhibit a telomere-mediated replicative senescence phenotype compared to humans (Akbar et al. 2001), and (iii) are genetically similar mouse breeds sterile housed for 600 days or more equivalent to human subjects with wide genetic variability and decades of exposure to multiple antigens from many different environments. These important differences between mice and man have led to the use of longer lived animal models, including baboons (Willis et al. 2014) and the rhesus macaque model (Cicin-Sain et al. 2011; Oxford et al. 2015), to interrogate the interaction of CMV and aging. Again, there can be issues with cross-species comparisons; as discussed in this paper by Ann Hill, the T cell responses generated to vaccines for RhCMV and HCMV in the respective hosts show distinct differences in the breadth and type of CD8 + T cell responses produced.
- In order to determine if the effect of CMV infection on global immune responses in aging is truly detrimental and requiring of medical intervention in older people will require far stronger evidence in order to convince the wider medical community of its need. For many physicians, CMV infection is not perceived as a major problem, because unless the patient is immune compromised or immune naïve, CMV-mediated disease is not commonly observed. Therefore, to provide a truly convincing argument in the future will require studies which also consider whether aging or putative lifelong CMV infection alters the quality of the immune response to the virus and then considers whether there are consequences for overall health. Many of the historical population studies performed to interrogate whether CMV is detrimental in aging in humans have relied on comparing total immune cell phenotypes with CMV serology relied on as a measure of determining infection history. Reliance on CMV IgG/IgM serology to identify CMV infectious history may be problematic, as anecdotal evidence has shown that there are discrepancies in the results seen from CMV serology test kits from different manufacturers, and CMV-specific T cells have been detected in CMV seronegative donors.
- Lastly, it is clear from murine studies that not only does CMV infection persist in different tissues but also the CMV-specific T cell response that is resident in these tissues can have distinct phenotype and functions (Verma et al. 2015). Within humans, the peripheral blood provides a mere snapshot of the immune response, comprising only 2% of the bodies’ total lymphocyte numbers (Blum and Pabst 2007). A recent study has identified CMV-specific CD8+ T cells in many different tissue sites in humans (via a collaboration with an organ transplantation body), and the results further suggest that CMV-specific T cells isolated from different tissues can also have distinct phenotype and functionality (Gordon et al. 2017). While it is not possible to access multiple tissue sites at one time in living human donors, there are possibilities through surgical collaborations to access immune cells from disparate tissue sites enabling comparison with immune cells isolated from peripheral blood. Studies have been published investigating the presence of CMV infection or CMV immune responses in the mucosal tissue (Clement et al. 2016), the lung via bronchoalveolar lavage fluid (Poole et al. 2015), and the bone marrow (Palendira et al. 2008). Interrogating the function and quality of tissue resident CMV-specific immune cells may help to inform our understanding of the frequency of CMV reactivation and dissemination and whether this is detrimental to an individual’s health.

Overall, it is clear there are still many questions regarding the impact of CMV-mediated immune evasion and manipulation of the immune system with aging that remain incompletely answered and that require further research.
